# A cascade of preventable complications following a missed femoral neck fracture after antegrade femoral nailing

**DOI:** 10.1186/1754-9493-7-16

**Published:** 2013-05-23

**Authors:** Lucas S McDonald, Frances Tepolt, Dominic Leonardelli, E Mark Hammerberg, Philip F Stahel

**Affiliations:** 1 Department of Orthopaedics, Naval Medical Center, San Diego, 34800 Bob Wilson Drive, San Diego, CA 92134, USA; 2 Department of Orthopaedics, Denver Health Medical Center, University of Colorado, School of Medicine, 777 Bannock Street, Denver, CO 80204, USA

**Keywords:** Femoral neck fracture, Femoral shaft fracture, Missed injury, Complication

## Abstract

**Background:**

Occult femoral neck fractures associated with femoral shaft fractures are frequently missed and may lead to adverse outcomes.

**Case presentation:**

A 46-year old female presented to our institution with increasing groin pain one month after antegrade intramedullary nailing of a femoral shaft fracture at an outside hospital. Radiographic evaluation revealed a displaced ipsilateral femoral neck fracture, adjacent to the piriformis starting point of the nail. A revision fixation of the femoral shaft and neck fracture was performed. The patient sustained a series of complications requiring multiple revision surgeries, including a total hip arthroplasty. Despite the cascade of complications, the patient had an uneventful long-term recovery, without additional complications noted at one-year follow-up.

**Conclusion:**

This case report illustrates the necessity of increased awareness with a high level of suspicion for the presence of associated femoral shaft and neck fractures in any patient undergoing antegrade femoral nailing. Arguably, the cascade of complications presented in this paper could have been prevented with early recognition and initial stabilization of the occult femoral neck fracture. Standardized diagnostic protocols include “on table” pelvic radiographs to rule out associated femoral neck fractures. The diagnosis must be enforced in case of equivocal radiographic findings, either by computed tomography scan or magnetic resonance imaging.

## Background

Femoral neck fractures associated with ipsilateral femur shaft fractures are uncommon injuries with a low incidence of 2% to 6% [1,2]. Occult femoral neck fractures can be challenging to diagnose and are missed in up to 30% of all cases [2,3]. The etiology of these associated injuries is either related to the primary traumatic event, or by a secondary (iatrogenic) intraoperative fracture induced by intramedullary femoral nailing [1,2]. Missed femoral neck fractures can displace over time and require additional surgical procedures, including a total joint replacement, which is associated with additional potential complications, including symptomatic limb length discrepancy and hip dislocation [4-6]. In this case report we describe a series of subsequent complications associated with a missed femoral neck fracture after femoral nailing and discuss potential root causes, preventability, and diagnostic strategies.

## Case presentation

**Figure 1:**
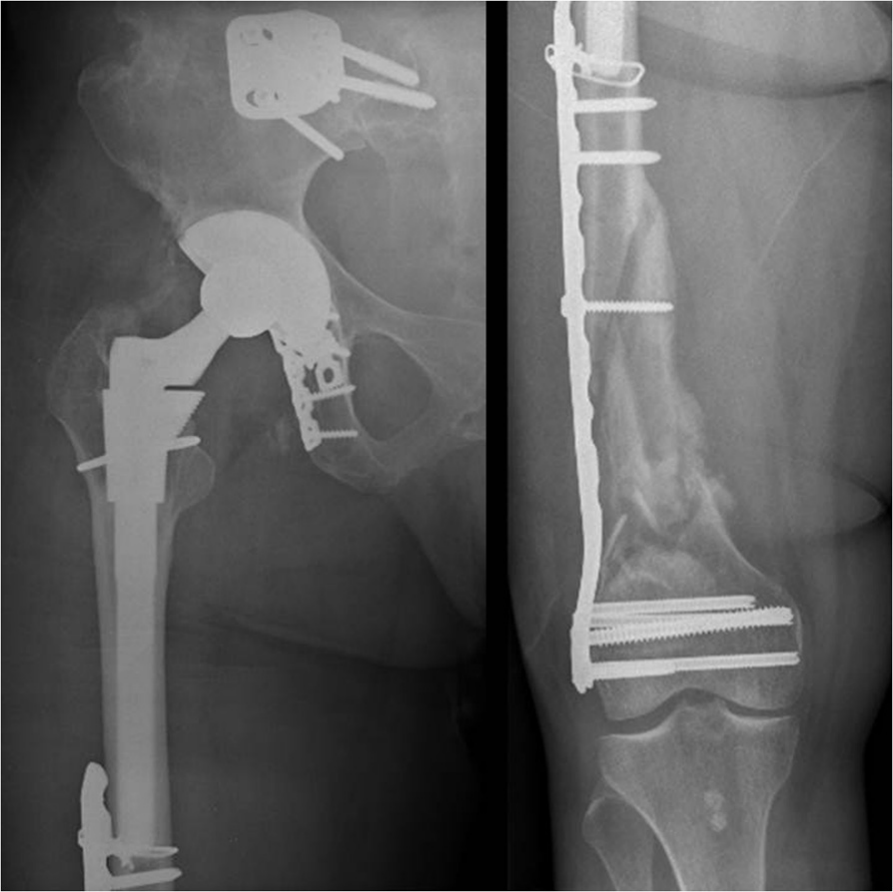
**Imaging of the right hip and femur of a 46-year old female patient at first presentation to our institution.** Plain radiographs demonstrate a right sacro-iliac joint fusion, right hip arthroplasty after acetabular fracture fixation, and bridge plating of a right distal femur fracture.

**Figure 2:**
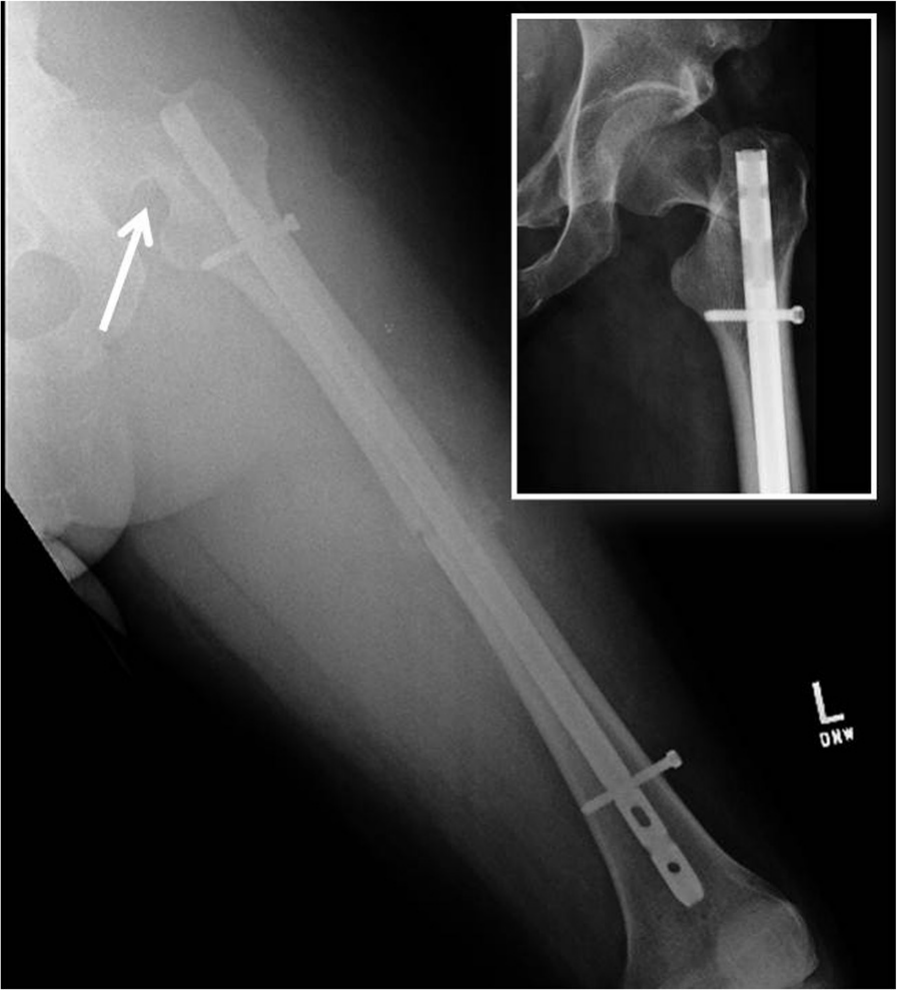
**Radiographs of the left femur in the same patient, demonstrating early signs of fracture healing of a transverse femur shaft fracture treated by intramedullary interlocking nail fixation.** The arrow and inset picture depict the displaced femoral neck fracture adjacent to the piriformis starting point of the antegrade nail.

**Figure 3:**
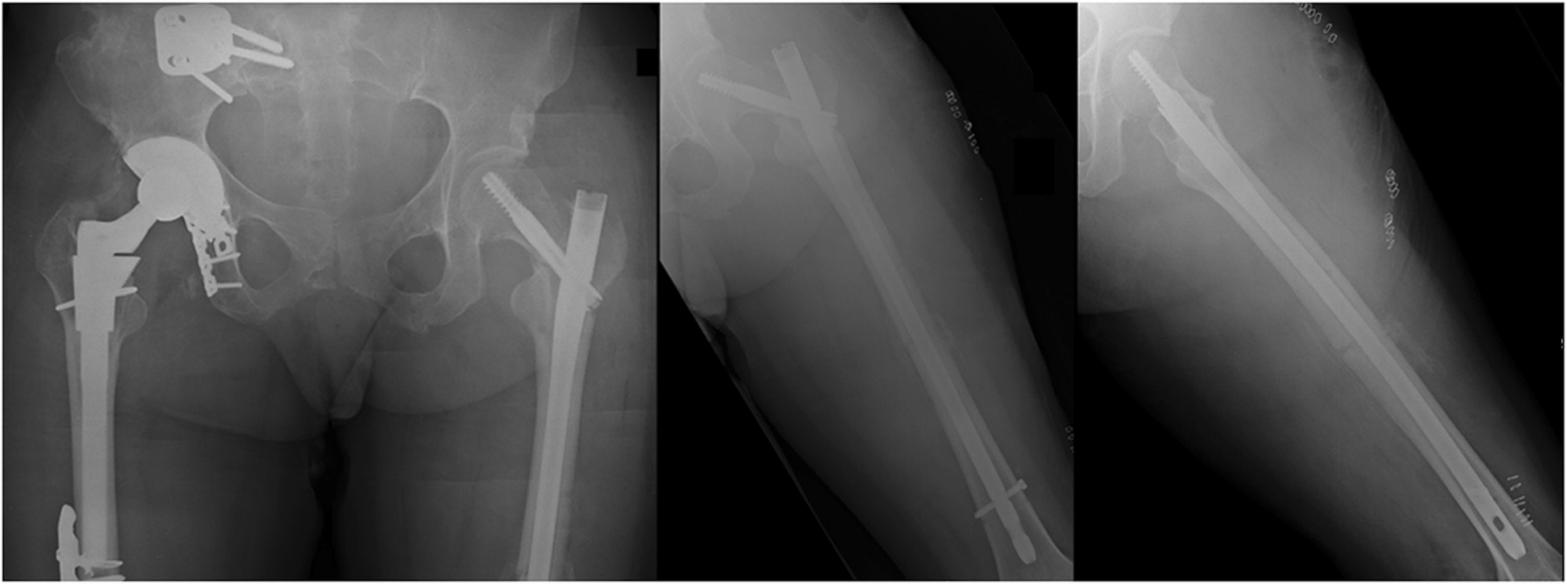
**Plain radiographs after the initial surgical revision with reduction and placement of a cephalomedullary nail for fixation of the femoral neck fracture and ipsilateral femur shaft fracture.** The fracture appears to be slightly malreduced in varus of the hip.

A 46-year-old female presented to the orthopaedic outpatient clinic of our level 1 trauma center one month after sustaining bilateral femur fractures in a motor vehicle accident. These injuries were treated at an outside hospital by locked plating of the right distal femur fracture, and by antegrade intramedullary interlocking nail fixation of the left femoral shaft fracture. She presented for a second opinion experiencing progressive left groin pain in the absence of an additional trauma, resulting in wheelchair dependency. The physical examination revealed healed surgical incisions on bilateral lower extremities, and impaired active and passive range of motion to the left hip secondary to significant pain. Neuromuscular examination of the distal left lower extremity was within normal limits. She had a leg-length discrepancy with shortening of the right leg secondary to multiple previous surgical procedures, which included a right total hip arthroplasty (THA) after acetabular fracture fixation, ipsilateral sacro-iliac joint fusion, and right-side distal femur plate fixation (Figure 1). The patient also demonstrated a right-sided foot drop. Radiographic evaluation revealed a displaced basicervical femoral neck fracture adjacent to the piriformis entry point of an antegrade femoral interlocking nail. This implant was effectively stabilizing a transverse midshaft femur fracture, with early signs of callus formation (Figure 2). After discussing all treatment options, the patient underwent closed reduction of the femoral neck fracture on a traction table, and revision fixation of both fractures was performed with a reamed cephalomedullary nail (Stryker Gamma 3™, Mahwah, NJ) through a greater trochanter starting point. Postoperative radiographs revealed an adequate cumulative tip-apex distance of <25 mm, however, the reduction appeared to be in slight varus of the hip (Figure 3).

**Figure 4:**
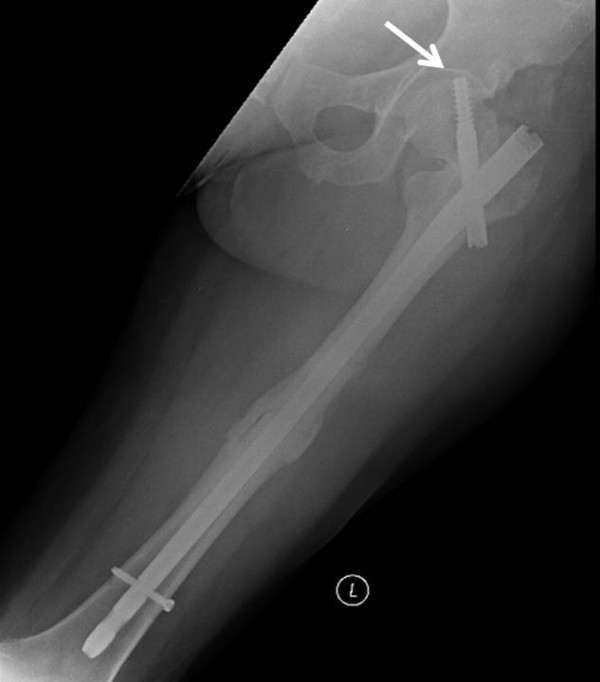
Failure of fixation of the cephalomedullary nail by “cut out” of the lag screw through the femoral head (arrow).

At two-week follow-up, the patient was ambulating with weight bearing as tolerated on crutches. All surgical incisions were healed and staples were removed. Six weeks later, she reported progressive worsening of left hip pain without additional hip trauma. She was no longer able to bear weight on the left leg and had resumed use of a wheelchair for 3 weeks. Physical examination demonstrated no indication of infection, but any motion of her left hip was extremely painful. Radiographs revealed a failure of fixation of the basicervical femoral neck fracture, with “cut-out” of the lag screw through the femoral head. The midshaft femur fracture was continuing to heal uneventfully (Figure 4).

**Figure 5:**
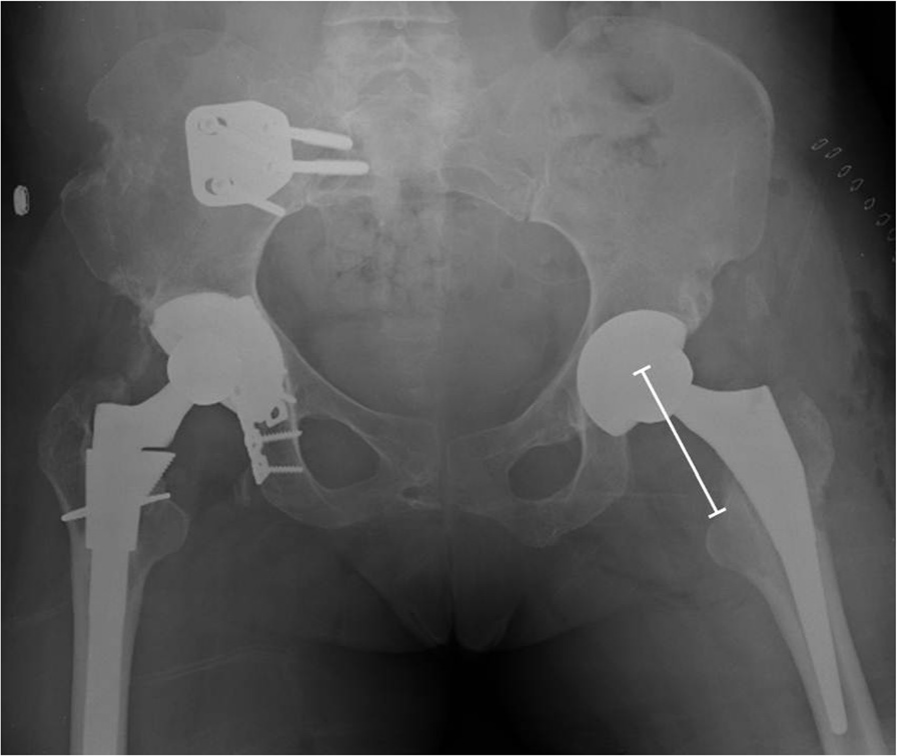
**Anteroposterior pelvic radiograph after the 2nd surgical revision of the left hip with a total hip arthroplasty.** Note the distance between femoral head center of rotation to the lesser trochanter.

**Figure 6:**
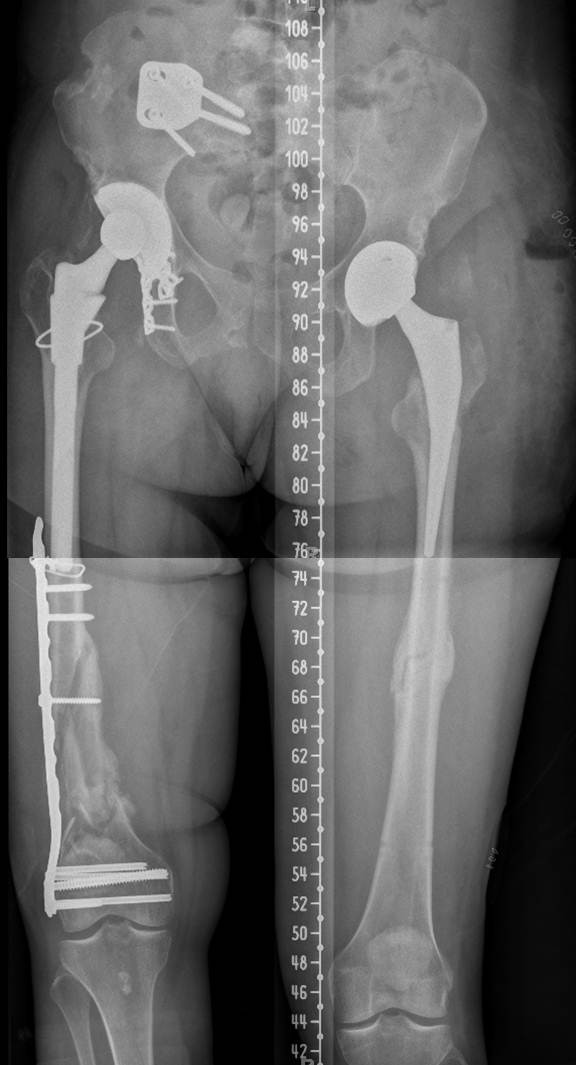
Full-length standing radiographs of bilateral femurs demonstrating a postoperative limb length discrepancy of 3.5 cm lengthening on the left side.

**Figure 7:**
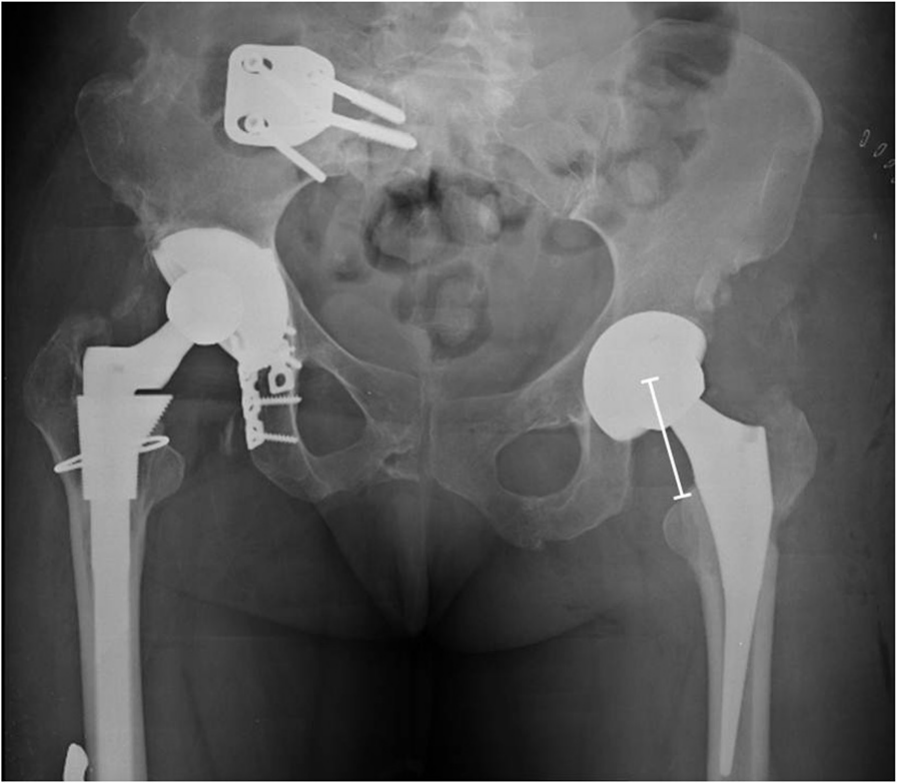
**Anteroposterior pelvic radiograph after the revision left total hip arthroplasty.** Note the decreased distance between femoral head center of rotation to the lesser trochanter, compared to Figure 5.

**Figure 8:**
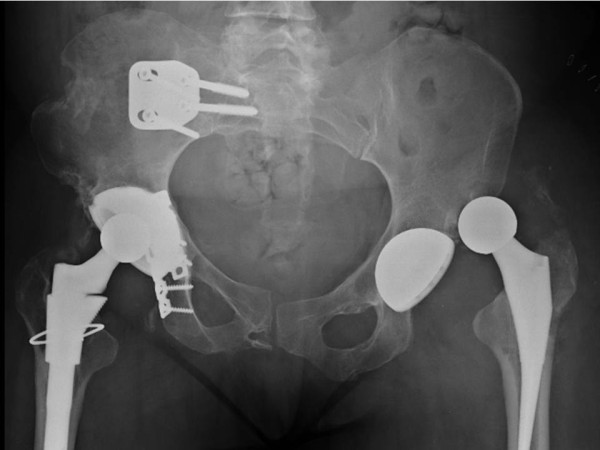
**Anterior hip dislocation following revision left total hip arthroplasty.** The dislocation was successfully managed by closed reduction and immobilization in a hip abduction brace (not shown).

Given the unsalvageable damage to the femoral head and acetabulum she was converted to a total hip arthroplasty (THA). The procedure was performed without complications, using non-cemented acetabular cup and press-fit stem components (Zimmer, Warsaw, IN). The stem of the arthroplasty appears to have been placed in slight varus position (Figure 5). On postoperative day one, the patient mobilized with physical therapy and noted a significant leg-length discrepancy, with the left leg approximately 3.5 cm longer than the right, as confirmed by full-length standing X-rays (Figure 6). The patient was offered a revision THA to partially correct the leg length discrepancy. She agreed and a revision THA was performed, including proximal femoral shortening and revision of acetabular and femoral components resulting in a near equal leg length (Figure 7). The patient subsequently sustained an acute anterior dislocation of her left THA revision at one week after discharge from the hospital, possibly related to the shortening procedure (Figure 8). The dislocation was successfully managed by closed reduction under general anesthesia. The intraoperative exam revealed that the hip was stable in full flexion and abduction, but unstable in extremes of extension or adduction. The THA dislocation was successfully managed in a hip abduction orthosis for 6 weeks. The patient had no further complications or adverse events, and presented with an acceptable outcome at one-year follow-up in our orthopaedic clinic.

## Discussion

This case report describes a series of complications secondary to a missed femoral neck fracture after antegrade femoral nailing in a 46-year old patient. The patient eventually achieved an acceptable functional outcome at one-year follow-up, despite multiple adverse events and re-operation. This unfortunate case illustrates the importance of an increased awareness combined with a high level of suspicion for the presence of associated femoral neck and shaft fractures. In the present case the initial index surgery was performed at an outside hospital and given a lack of access to initial radiographs, it is impossible to speculate whether the femoral neck fracture occurred as an iatrogenic complication during the intramedullary nail procedure, or whether this was a trauma-related injury missed during the initial assessment. Regardless of the exact root cause, this case presents a series of adverse events and preventable complications which are ultimately due to the initially missed femoral neck fracture, which would have been amenable to primary stabilization as part of the index procedure. We will further discuss the individual specific complications in relation to the pertinent peer-reviewed literature.

### Missed femoral neck fracture, with associated femur shaft fracture

Concomitant fractures of the ipsilateral femoral neck and femoral shaft are uncommon injuries, most often seen in young adult patients and secondary to high-energy mechanisms, including motor vehicle accidents or falls from heights [1,2,7-11]. Ipsilateral femoral neck fractures occur in only 2% to 6% of all patients with femoral shaft fractures [1,8-14]. Longitudinal compression is most commonly responsible for this fracture pattern with the femoral shaft absorbing much of the energy before causing a vertical, minimally displaced, and easily masked femoral neck fracture [1,2,8,15-17]. When first described about 60 years ago, the rates of missed associated femoral neck and shaft fractures were between 50% and 80%, although this has improved to as low as 11% with heightened awareness, improved radiographic assessment, and standardized protocols at regional trauma centers [1,2,7-10,13,18-20]. Complications of this injury, particularly if the femoral neck fracture component is missed, include additional surgical interventions [20], nonunion or malunion [9,16-18,21], and avascular necrosis [8,9,18,22]. Avascular necrosis occurs in approximately 5% of patients with this dual injury pattern, although published rates range from 4% to 22% with the exact rate unclear due to frequent lack of long-term follow-up [1,2,9,10,12].

Establishing an accurate diagnosis remains challenging, and early clinical exam may be limited by distracting injuries or obtunded patients [8,9]. Many authors recommend an anteroposterior pelvis radiograph and imaging of the entire femur in any patient with high-energy trauma and femoral shaft fractures [3,7,9,18]. Imaging of the femur should include dedicated anteroposterior internal rotation and lateral views of the hip performed prior to initiation of surgical treatment [1,2,7-9,11,18-20]. Many trauma patients are evaluated with computed tomography scans of the abdomen and pelvis, providing orthopaedic surgeons a “free look” at the femoral neck [1,2,9].

Tornetta *et al*. published the results of instituting an institutional protocol to evaluate for femoral neck fractures in all patients with femoral shaft fractures. This institutional protocol includes preoperative anteroposterior internal rotation plain radiographs of the hip, and a fine cut (2-mm) computed tomographic scan through the femoral neck as part of the initial trauma scan and an intraoperative fluoroscopic lateral radiograph of the hip prior to fixation of the femoral shaft. Additionally, anteroposterior and lateral radiographs of the hip are obtained post femoral shaft fixation but prior to extubation, evaluating the femoral neck for fracture. At follow-up, anteroposterior and lateral radiographs are again obtained and the patient is questioned about hip pain with any concerning findings sent for repeat computed tomography. The results appear to be encouraging, with a significantly improved detection rate and a reduction in delay of diagnosis by more than 90% [3].

### Iatrogenic femoral neck fractures associated with piriformis starting point

The pioneer of femoral nailing, Gerhard Küntscher, warned about iatrogenic femoral neck fractures and recommended a lateral-based greater trochanter entry point [23]. The piriformis fossa entry point for antegrade femoral nails was later described by Winquist in the 1980s [22]. Subsequently, multiple authors have reported on the potential for iatrogenic femoral neck fractures associated with a piriformis starting point, with a 1% incidence [24]. Contributing factors include: the precise anatomic location of the entry point [25-27]; the number of trial drill holes [24,28]; the orientation of the awl and intramedullary nail at insertion [24]; and the diameter of the intramedullary nail [26]. The general consensus in the peer-reviewed literature is that careful attention must be applied to the precise location of the piriformis entry point, the correct orientation of the opening awl, and the minimization of the number of trial drill holes [24-28].

In the present case report, the question of whether the root cause of this patient’s displaced femoral neck fracture was iatrogenic or related to the primary trauma remains purely speculation. Management options for revision surgery include: (1) leaving the current femoral shaft implant in place and attempting a closed or open reduction with lag screw fixation around the femoral implant; (2) removing the antegrade femoral nail and applying a sliding hip screw/plate device for the basicervical fracture, in conjunction with and revision femoral fixation by a retrograde nail; or (3) revision fixation of both fractures with a single device, such as a cephalomedullary nail, as performed in the present case (Figure 3).

### Failure of proximal femur fixation

Complications have been associated with many types of implants used in the fixation of ipsilateral femoral neck and shaft fractures, including intramedullary devices, sliding hip screws and cannulated screws [29-32]. Among the known complications are nonunion at one or both sites [32] and failure of fixation [29-31,33]. Types of mechanical failure associated with sliding hip screws in particular include loss of the implant’s dynamic action and disassociation of the plate from the femur [30]. When considering the specific implant failure mode of femoral head cutout, as seen in this patient, most literature is based on research performed on intertrochanteric fractures [29,31]. In the literature, femoral head cut-out following the fixation of intertrochanteric or pertrochanteric fractures has been associated with screw placement in the upper one-third of the femoral head [31], or a cumulative tip-apex distance greater than 25 mm [29].

The tip-apex distance as described by Baumgartner *et al*. is the sum of the distance from the tip of the lag screw to the apex of the femoral head on an anteroposterior radiograph and this same distance on a lateral radiograph [29]. Their study evaluated 198 pertrochanteric fractures stabilized with sliding hip-screw devices including side-plate and intramedullary devices. They discovered a direct relationship between an increased tip-apex distance and risk of lag screw cut out with a 2% failure rate with tip-apex distances less than 30 mm and no failures with tip-apex distances less than 25 mm. Additionally they found the greatest rates of cutout for lag screws placed in the posterior-inferior and anterior-superior zones and higher rates of failure for unstable fracture patterns [29].

In the present case, we opted to revise the piriformis nail to a trochanteric entry cephalomedullary nail with a femoral neck lag screw. Despite adequate cumulative tip-apex distance of less than 25 mm, the reduction is slight varus of the hip (Figure 3) may have contributed as a root cause for failure of fixation (Figure 4). Additionally, the patient’s early weight bearing before radiographic and clinical healing likely contributed to failure of fixation.

### Limb length discrepancy after THA

The incidence of limb length discrepancy (LLD) following THA is challenging to define due to its multifactorial cause and the discrepancies that often exist between subjective and objective findings [4,34-36]. Root causes of LLD are frequently related to acetabular or femoral component positioning, although most commonly result from femoral component lengthening [4]. Component malposition can indirectly cause LLD limb length discrepancy secondary to intra-operative correction with soft tissue tightening or release [35]. Many patients perceive some degree of leg length inequality early in the post-operative period, which often resolves with time and physical therapy. The surgeon must distinguish between functional (perceived) and “true” (symptomatic and radiographically confirmed) LLD [35,36]. True LLD can have potentially serious complications, including persistent pain, hip instability, and paresthesias. Revision THA is a treatment consideration when surgically correctable causes of LLD are identified [34,35].

In the present case report, our patient demonstrated a radiographically confirmed LLD of 3.5 cm after the initial joint replacement procedure. Root causes in the present case include the complex nature of the patient’s previous hip and pelvic trauma with shortening of the contralateral femur during the previous THA (Figure 6).

### Hip dislocation after revision THA

The reported rate of dislocation following primary THA ranges from less than 1% to almost 10% [37,38]. Preceding surgical procedures represent a significant risk factor predisposing to instability, and has been shown to double the risk of a postoperative dislocation [6,37-39]. The published dislocation rate following revision THA ranges from 7% to 9% [5,6]. The increased risk of dislocation after revision surgery relates to the extent of soft tissue damage and muscular weakness. This becomes particularly relevant if the revision surgery is performed to correct recurrent instability [6]. Other risk factors for dislocation include use of a posterior approach, trochanteric nonunion, smaller femoral head component size, the use of non-elevated rim liners, and the early postoperative period of less than 3 months after surgery [5,6,37-39]. Hip dislocations early in the postoperative period are best avoided with physical therapy protocols that include appropriate range of motion restrictions and other precautionary measures [39]. If a dislocation occurs, the hip should be reduced and immobilized for six to twelve weeks by spica casting, bracing or knee immobilization [37]. The slight varus malposition of the stem may have additionally contributed to the repeat hip dislocation in this case (Figure 8). Repeat dislocations may necessitate revision surgery including component re-orientation, posterior acetabular wall extension (for posterior dislocations), and trochanteric advancement procedures [37]. The success rate of revision THA for chronic dislocation has been described to be as low as 50% [37].

## Conclusion

This case report highlights multiple unfortunate events and preventable complications in a young patient treated for bilateral femur fractures after a motor vehicle accident. The sentinel complication consists of a missed femoral neck fracture during the initial work-up or at the time of antegrade femoral fixation. This case illustrates the importance of an adequate pre-operative radiographic workup, including internally rotated anteroposterior and lateral plain radiographs of the hip for visualization of the femoral neck, both prior to and after completion of antegrade femoral nailing. The availability of pre-operative computed tomography (CT) scans obtained during the full-body trauma work-up allows for the orthopaedic surgeon to scrutinize the images for the presence of an occult, nondisplaced femoral neck fracture. In addition, fluoroscopic views of the femoral neck and “on table” plain radiographs should be performed as a standard protocol, prior to patient awakening and extubation [2,3]. The present case report serves as a reminder to orthopaedic trauma surgeons, and to the next generation of surgeons in training, to carefully analyze all pre-/intra-/and post-operative radiographs for associated injury, and to have a low threshold for further the preoperative evaluation of suspected concomitant femoral neck and shaft fractures with adjunctive diagnostic strategies including thin-cut CT scans and magnetic resonance imaging (MRI) when radiographic findings are equivocal.

## Consent

Written informed consent was obtained by the patient described in this manuscript for publication of the case report, including all radiographs and images shown in the figures.

## Abbreviations

CT: Computed tomography; LLD: Limb length discrepancy; MRI: Magnetic resonance imaging; ORIF: Open reduction and internal fixation; THA: Total hip arthroplasty.

## Competing interests

The authors declare no competing interests related to this manuscript. The views expressed in this article are those of the authors and do not reflect the official policy or position of the Department of the Navy, Department of Defense, or the United States Government.

## Authors’ contributions

PFS and MH designed the case report. LSM and FT wrote the first draft of the manuscript. DL systematically edited the manuscript. All authors critically revised this paper, and read and approved the final version of the manuscript.
